# Air quality standards and WHO's guidance on particulate matter measuring 2.5 μm (PM2.5)

**DOI:** 10.2471/BLT.23.290522

**Published:** 2024-12-03

**Authors:** Yevgen Nazarenko, Devendra Pal, Sanjeev Dwivedi, Parisa A Ariya

**Affiliations:** a Department of Environmental and Public Health Sciences, University of Cincinnati, Cincinnati, United States of America.; b Department of Atmospheric and Oceanic Sciences, McGill University, 805 Sherbrooke Street West, Montreal, QC H3A 0B9, Canada.; c Meteorological Centre, Ministry of Earth Sciences, Bhubaneswar, India.

The World Health Organization’s (WHO) normative guidance on ambient air quality^1^ is based on the evidence from epidemiological, health and exposure studies regarding the harms associated with certain mass concentrations of airborne particulate matter, expressed as PM_2.5_. The definition of PM_2.5_ is the mass concentration of particles smaller than approximately 2.5 μm. These guidelines are a critical reference point for jurisdictions developing or revising their ambient air quality standards. However, the guidance does not cover the harmonization of averaging methods for concentrations measured during data aggregation or for handling exceedances of PM_2.5_ levels. Yet, harmonization is essential to ensure the accurate collection of comparable pollution data globally, as variations in measurement approaches can obscure true ambient pollution levels.^2^ Furthermore, the guidelines do not account for the fact that the particle-mass-based PM_2.5_ metric does not consider physicochemical characteristics of airborne particles such as size, chemical composition or the bioavailability of potentially harmful elements in the particles.^3^ Critically, the metric does not reflect the particle number concentration (PN) of differently sized particles, including ultrafine particles, and thus misses the full scope of health-harming particulate air pollution.

While both fine and ultrafine particles are included in the PM_2.5_ metric, PM_2.5_ mass comes mostly from fine particles. However, most particles in the typical ambient environment are ultrafine particles, which are defined as less than approximately 100 nm in size. The mass of ultrafine particles is negligible compared to fine particles in PM_2.5_; however, most health studies only consider the total mass of airborne PM_2.5_ particles.^4^ When PM_2.5_ is higher than 5 µg/m^3^, the mass concentration does not correlate well with the particle number of ultrafine particles,^5^^–^^7^ and therefore control measures aiming to reduce high PM_2.5_ levels might not reduce ultrafine particles. However, a good correlation exists between particle number and PM_2.5_ when the concentrations are below approximately 10 000 particles/cm^3^ and 5 µg/m^3^, respectively, suggesting that meeting the WHO recommendation for a maximum 5 µg/m^3^ of PM_2.5_ will likely keep ultrafine particulate air pollution within an acceptable range. However, most countries are far from achieving such low ambient air pollution.^8^

Evidence suggests that short-term exposure to ultrafine particles is associated with respiratory symptoms and systemic inflammation, and can affect heart rate and blood pressure.^9^ Furthermore, long-term exposure to ultrafine particles is associated with increased mortality, especially cardiovascular and lung-related mortality, and several types of morbidity, such as ischaemic heart disease.^10^^,^^11^ As the health effects of ultrafine particles are better associated with their number density rather than mass, monitoring and analysing the number density of particles smaller than either 2.5 μm or 100 nm, in addition to PM_2.5_ are required to measure the human exposure to and harm from ultrafine particles.

A number-based metric directly indicates the number of ultrafine particles in ambient air, even when using a particle size range wider than ultrafine particles because by number, most particles in ambient air are ultrafine. Therefore, we propose a complementary number-based metric to use in parallel with PM_2.5_, named PN_2.5_, to reflect the number density of particles within the PM_2.5_ mass fraction of ambient aerosols.

The research community must collaborate with government agencies to develop a standard approach for ambient ultrafine particle monitoring, to enable comparison of monitoring data from different jurisdictions when using different analytical equipment. WHO, with participation of the research community and standardization bodies such as the International Organization for Standardization, needs to develop standardized measurement techniques, data aggregation and analysis methods, and validation approaches, which should be added to WHO normative guidance. In this process, factors such as topographical and meteorological known sources of pollution and population distribution should be considered. 

We support the introduction of the novel PN_2.5_ metric that can be included in the existing mass-based PM_2.5_ ambient air quality regulations where the PM_2.5_ metric is already in use. The definition of the new PN_2.5_ metric, and the data collection, aggregation and reporting methods will remain the same as defined for the PM_2.5_ ambient air quality standards, with the exception that the PN_2.5_ will report the number of aerosol particles per cm^3^ of air up to the same particle size cut-off as currently used for the PM_2.5_ metric. This approach makes introducing the PN_2.5_ metric straightforward, without significant revisions to the existing PM_2.5_ ambient air quality standards.

However, challenges exist in incorporating the number-based PN_2.5_ metric in the WHO ambient air quality guidelines. Few government agencies currently monitor airborne ultrafine particles, neither using a particle number nor a mass metric. Nonetheless, a few studies have included short- or medium-term monitoring of ultrafine particles in ambient air in selected locations.^5^^–^^7^ In each case, the location had a significant impact on the accumulation and dissipation of ultrafine particles, as these particles are not transported over long distances and are typically found at elevated concentrations near their sources.^5^^,^^12^ Roadside measurements are useful, as traffic is a major source of ultrafine particles.^13^ Urban canyons and topography independently affect the concentration of ambient ultrafine particles at different proximity to sources.^12^ Ultrafine particle number concentrations vary seasonally and annually, showing more pronounced changes with meteorological conditions than PM_2.5_.^5^^,^^7^ As the number-based concentration of ambient ultrafine particles varies more than the PM_2.5_ concentration, both spatially and temporally,^5^ we favour higher temporal resolution such as with an hourly standard. 

Several robust and economical devices, such as diffusion-charging-based ultrafine particle monitors, are available from various manufacturers. Such devices may be suitable for widespread deployment at ambient air-quality monitoring stations. Optical-based and other monitors are promising and will likely serve worldwide in the future. The WHO guidelines may specify the requirements for the measurement techniques, methods and instruments suitable for worldwide ultrafine particle monitoring to ensure consistency and comparability.

Further research is needed to provide sufficient evidence for WHO to re-evaluate the aerosol particle concentration metrics used in the next iteration of air quality guidelines. With the incorporation of PN_2.5_ or PN_0.1_ into WHO’s ambient air quality normative guidelines, averaging methods and the rules regarding exceedances with both PM_2.5_ and PN_2.5_ or PN_0.1_ should be harmonized. Such harmonization will help reveal ambient air pollution of ultrafine particles, and allow accurate comparisons and research based on the global data relying on these monitoring methods.^2^
